# Predictors of Anxiety and Depression in Medical Professionals During the Time of COVID-19 Outbreak

**DOI:** 10.1017/dmp.2021.67

**Published:** 2021-03-08

**Authors:** Zhengjia Ren, Zhongyao Xie

**Affiliations:** 1 Department of Clinical Psychology, The Third Affiliated Hospital of Chongqing Medical University, Chongqing, China; 2 China University of Political Science and Law, School of Sociology, Beijing, China

**Keywords:** medical professionals, anxiety, depression, social media, COVID-19

## Abstract

**Objectives:**

The aim of this study was to investigate the influences of sociodemographic data, mental disorder history, confusion and somatic discomfort triggered by social media on anxiety and depression symptoms among medical professionals during the coronavirus disease 2019 (COVID-19) outbreak.

**Methods:**

A total of 460 participants completed online questionnaires that included sociodemographic data, mental health disorder history, an assessment of confusion and somatic discomfort triggered by social media, and psychological disturbance. Hierarchical linear regression model was adopted to analysis the data.

**Results:**

The hierarchical linear regression model was able to explain 41.7% of variance in depression symptoms, including comorbidity with 1 mental disorder (B = 0.296; *P* < 0.001), confusion (B = 0.174; *P* < 0.001), and somatic discomfort (B = 0.358; *P* < 0.001) triggered by social media. The hierarchical linear regression model was able to explain 41.7% of variance in anxiety symptoms, including sex (B = -0.08; *P* < 0.005), comorbidity with 1 mental health disorder (B = 0.242; *P* < 0.001), confusion (B = 0.228; P < 0.001), and somatic discomfort (B = 0.436; *P* < 0.001) triggered by social media.

**Conclusions:**

These results suggest that it is important to provide adequate psychological assistance for medical professionals with mental health problems in COVID-19 to buffer the negative impact of social media.

The outbreak of coronavirus disease 2019 (COVID-19) has brought significant impact to the mental health of the public. According to the China Mental Health Survey, a national epidemiological survey with 32,552 respondents, the prevalence of depressive disorder in general population of China is 6.8%. As for anxiety disorder, the prevalence is 7.6%.^1^ However, according to a survey after the first month of COVID outbreak, moderate-to-severe stress, anxiety, and depression were noted in 8.1%, 28.8%, and 16.5%, respectively, in general public.^2^

Medical professionals are usually exposed to high occupational stress, which makes them more vulnerable to mental disorders such as anxiety, depression, and occupational burnout than general populations.^3^ Previous studies have shown that medical workers experience a relatively high level of psychological disturbances, and the levels of stress, anxiety, and depression among this population seem to be much more severe than expected. In a systematic review, the estimated prevalence of depression or depressive symptoms among resident physicians was 28.8% (ranging from 20.9% to 43.2%).^4^ Another study in England found that anxiety and depressive symptoms were prevalent in 29% and 27% senior health professionals, respectively.^5^ Researchers in China showed that the prevalence of anxiety symptoms among physicians was 25.5%, and the prevalence of depressive symptoms reached 28.1%.^6^

In addition to the stress from their daily life and work, medical workers usually encounter more life-threatening stress than general populations. One previous study found that the prevalence of psychiatric morbidity was approximately 75% among health workers during the outbreak of severe acute respiratory syndrome (SARS).^7^ In 2019, an outbreak of a novel coronavirus pneumonia (COVID-19) occurred in China. More than 1700 health workers in China have been infected by COVID-19. Facing this large-scale infectious public health event, medical staff are under both physical and psychological pressure. Hospitals are usually areas with a high incidence of infection. Medical staff not only have to face their own negative emotions, such as fear and worry about the disease, but also need to face all kinds of clinical work during the epidemic. Undoubtedly, these pressures increase the mental and psychological burden of medical staff.

During the peak of the epidemic, all Chinese people experienced a variety of worries, fears, and anxieties in the face of the growing epidemic and the overwhelming confusion of the daily news. For the medical staff, the confusing news undoubtedly increased their fear and worry, such that they worried every day about issues, such as whether the patients they come into contact with are infected, being infected themselves, and transmitting the virus to their family.

Somatic symptoms are a common physical response to stressful information, and previous research claimed that somatic complaints were significantly associated with stressful life incidents and information.^8,9^ Emotional distress and arousal toward stressful information or incidents frequently give rise to bodily sensations. Thus, the response is associated with increased activity of the sympathetic nervous system, resulting in many bodily changes, including sweating, pounding heart, tachycardia, shaking, muscle tension, shortness of breath, dizziness, etc.^10^ It is proposed that heightened somatic and physiological conditions induced by stressful events may increase the total amount of time that stress has a “wear and tear” effect on the human body,^11^ while the extent of somatic discomfort triggered by social media and the impact of social media on the mental health and well-being of medical professionals is largely unreported.

Research on the influences of social media on the psychological well-being of the population has increased recently. Social media has become fundamental in the way many people and organizations communicate and share opinions, ideas, and information. Previous researchers claimed that exposure to information loaded with feelings of confusion, uncertainty, anxiety, fearful, desperation, and sadness and negative information usually leads to less trust in health recommendations and increased psychological disturbances.^12,13^ The impact of the Internet, particularly social media, on psychological disturbances is a topic of growing interest that has drawn national attention to this topic. To some extent, social media’s influence on psychological disturbance should be considered a public health problem, and how public health approaches might be used to address this influence is a relevant issue. In this article, we discuss the role of social media on psychological disturbance among medical workers from a public health perspective. At the same time, medical personnel often have a higher prevalence of mental health disorders. Consequently, we examined Chinese physicians’ anxiety and depressive symptoms as well as evaluated their mental health disorder history and social media use during the outbreak of COVID-19.

## Methods

### Ethics Statement

The study protocol was approved by the Research Ethics Committee of China University of Political Science and Law. All the participants read the purpose statement of the investigation and consented to participate in this research.

### Participants and Sampling

This study used a cross-sectional survey. Data were collected from February 9 to February 20 through an online platform. A total of 460 medical professionals completed the questionnaire through self-administered online anonymous questionnaires.

### Measurement

#### Demographic Information

Sociodemographic information was collected, including sex, age, education level, income, ethnic minorities, marital status, and religious belief.

#### Medical History of Mental Health Disorders

Participants responded to the following prompt regarding their medical history. Please select the disease you have been diagnosed with from the list below: depression, anxiety, bipolar disorder, schizophrenia, obsessive disorder, psychosomatic disorder, etc.

#### The Extent to Which You Have Received Information About COVID-19

Participants rated their feelings of confusion and somatic discomfort on a scale from 1 (not at all) to 10 (very strong).

### Psychosocial Measures

#### Generalized Anxiety Disorder Scale

The *Generalized Anxiety Disorder (*GAD-7) scale was used to measure generalized anxiety symptoms in the past 2 wk on a 4-point scale from 0 (not at all) to 3 (nearly every day).^14^ The total scores on the GAD-7 range from 0 to 21, and higher scores indicate more symptoms. The measure demonstrated adequate internal consistency, with Cronbach’s alpha = .95.

#### Patient Health Questionnaire

Depression was assessed with the Patient Health Questionnaire (PHQ-9). The PHQ-9 is a continuous measure of the frequency of symptoms of depression in the past 2 wk.^15^ Each of the 9 items is rated on a scale from 0 (not at all) to 3 (nearly every day). The total PHQ-9 score ranges from 0 to 27, and higher scores indicate severe symptoms. The validity and reliability of the PHQ-9 have consistently been verified in different studies of the Chinese population. In the current study, the Cronbach’s alpha of the scale was 0.95.

### Statistical Analysis

All statistical procedures were performed using the Statistical Package for Social Sciences Version 22.0 for Windows. Descriptive analysis was carried out for sociodemographic data, mental health disorder history, social media, and anxiety and depression. The prevalence of anxiety and depressive symptoms was calculated and compared with sociodemographic characteristics, history of mental health disorder, feelings of confusion triggered by social media exposure, and somatic discomfort triggered by social media exposure. Hierarchical regression was used to examine the potential additive effect of a history of mental health disorders, feelings of confusion triggered by social media exposure, and somatic discomfort triggered by social media exposure on depression and anxiety. Hierarchical regression involves the prediction of variance in a continuous dependent variable by blocks of related independent variables to examine the additive and unique effects of different variables. Sociodemographic variables were entered as covariates in the first block; history of mental health disorders, confusion triggered by social media exposure, and somatic discomfort triggered by social media exposure were entered in the second block. Adjusted odds ratios (ORs) and 95% confidence intervals (CIs) for each variable were calculated. For all comparisons, differences were tested using 2-tailed tests, and *P* values less than 0.05 were considered statistically significant.

## Results

### Basic Characteristics

Data collected from 460 (131 male and 329 female) medical professionals were analyzed. For marriage status, most of the participants were married (264/460; 57.4%), and 38.5% of them were single (177/460). Most of them claimed that they did not have any religious belief (388/460; 84.3%). A total of 94.1% of medical professionals were from the Han ethnic group (433/460), and 5.9% of the participants were from ethnic minority groups (27/460). Other detailed characteristics of the included patients are summarized in Table 1.

**Table 1.** Demographics information of the participants

Participants	Variables	*N* = 460
Gender	Women	329 (71.5%)
	Men	131 (28.5%)
Age (y)	≤30	221 (48.1%)
	31< Age ≤40	130 (28.3%)
	41< Age ≤50	84 (18.3%)
	Above 51	25 (5.4%)
Income	Below 3000 RMB	111(24.2%)
	3001-5000 RMB	124(27%)
	5001-7000 RMB	100 (21.7%)
	7001-1000 RMB	61 (13.3%)
	10001 RMB above	64 (13.9%)
Marital status	Single	177(38.5%)
	Married or cohabit	264 (57.4%)
	Divorced or other	19 (4.1%)
Religion	No	388(84.3%)
	Yes	72 (15.7%)
Ethnicity	No	433 (94.1%)
	Yes	27 (5.9%)
Education	Associate degree	91 (19.8%)
	University	261(56.7%)
	Master’s degree or above	108 (23.5%)
Mental health disorder history	No history of mental health disorder	324 (70.4%)
	One mental health disorder	84 (18.3%)
	Comorbidity with one mental health disorder	31 (6.7%)
	Comorbidity with two mental health disorder	21 (4.6%)

For mental health disorder history, most of the participants reported no history of mental health disorder (324/460; 70.4%); 18.3% of them suffered from 1 mental health disorder (84/460); 6.7% reported comorbidity with 1 mental health disorder (31/460); and 4.6% reported comorbidity with 2 mental health disorders (21/460).

To specifically examine our 3 hypotheses, hierarchical regression analyses were used to examine participants’ reports of (1) the history of mental health disorders, (2) confusion triggered by social media, and (3) somatic discomfort triggered by social media. In these regression analyses, the covariates age, education, religion, marriage situation, and income level were entered into Step 1; mental health history and comorbidity were entered into Step 2; confusion triggered by social media was entered into Step 3; and somatic discomfort triggered by social media was entered into Step 4. For these analyses, all variables were centered.

Hypothesis 1 was supported, indicating that participants’ mental health disorders were positively associated with depression and anxiety. In other words, participants who reported having mental health disorders were more likely to report depression and anxiety.

Hypothesis 2 was that participants’ increased feelings of confusion triggered by social media would be positively associated with anxiety or depression.

Hypothesis 3 was that participants’ increased somatic discomfort triggered by social media would be positively associated with anxiety or depression.

When considering covariates and mental health comorbidity, both of the variables were positively related to anxiety or depression. In other words, participants who reported more comorbidity with mental health disorders and more negative feelings triggered by social media also reported severe anxiety or depression (see Tables 2 and 3). The first step included age, education level, marriage, sex, major, marriage status, and income level. This model was significant, accounting for 1.7% of the variance in anxiety. Mental health comorbidity was added in the second step and was a significant independent predictor of anxiety, and this model accounted for 20.7% of the variance in anxiety. Confusion triggered by social media was added in the third step and was a significant independent predictor of anxiety, and this model accounted for 35.8% of the variance in anxiety. Somatic discomfort triggered by social media was added in the last step and was a significant independent predictor of anxiety, and this model accounted for 49.8% of the variance in anxiety.

**Table 2.** Predictors of depression

	B(SE)	*ˇB*	t	*P*-Value	Adjusted R^2^
Step 1					
Sex	−.765(.417)	−.068	−1.835	.067	
Age	−.241(.267)	−.045	−.901	.368	
Education	−.221(.267)	−.033	−.828	.408	
Income	.175(.156)	.050	1.125	.261	
Ethnic minority	−.018(.783)	−.001	−.023	.982	
Religion	−.022(.515)	−.002	−.043	.966	
Marriage	−.230(.441)	−.025	−.521	.603	
Step 2					
Comorbidity of mental health disorder	1.848(.242)	.296	7.649	>.00	0.217 ΔR^2^ = .203
Step 3					
Confusion induced by social media	.356(.086)	.174	4.160	>.00	0.310 ΔR^2^ = .093
Step 4					
Somatic discomfort induced by social media	1.023(.121)	.358	8.458	>.00	0.417 ΔR^2^ = .093

**Table 3.** Predictors of anxiety

	B(SE)	ˇB	t	*P*-Value	Adjusted R^2^
Step 1					
Sex	−.750 (.320)	−.080	−2.344	.019	
Age	−.005 (.205)	−.001	−.025	.980	
Education	−.008 (.205)	−.001	−.038	.970	
Income	.081 (.120)	.028	.676	.499	
Ethnic minority	−.039 (.601)	−.002	−.065	.948	
Religion	−.097 (.395)	−.008	−.245	.807	
Marriage	.335 (.338)	.044	.990	.323	
Step 2					
Comorbidity of mental health disorder	1.263 (.185)	.242	6.815	>.00	0.207 ΔR^2^ = .189
Step 3					
Confusion induced by social media	.390 (.066)	.228	5.941	>.00	0.358 ΔR^2^ = .150
Step 4					
Somatic discomfort induced by social media	1.042 (.093)	.436	11.225	>.00	0.498 ΔR^2^ = .138

Regarding depression, the first step included age, education level, marriage, sex, major, marriage status, and income level. This model was not significant, accounting for 1.3% of the variance in depression. Mental health comorbidity was added in the second step and was a significant independent predictor of anxiety, and this model accounted for 21.7% of the variance in depression. Confusion triggered by social media was added in the third step and was a significant independent predictor of depression, and this model accounted for 31% of the variance in depression. Somatic discomfort triggered by social media was added in the last step and was a significant independent predictor of depression, and this model accounted for 40.4% of the variance in depression.

## Discussion

Medical professionals have a heavier burden during the COVID-19 outbreak, and they also encounter similar or even more stress than the general population due to exposure to COVID-19-infected citizens. The stresses caused by the COVID-19 outbreak, such as a heavy workload, worries about being infected, a lack of social interaction, and worries about contracting the virus and transmitting it to family members, may make medical professionals anxious and depressed.

The current study showed that a history of mental health disorders has a significant positive correlation with both anxiety and depression, indicating that mental health disorder medical history is an important factor that affects medical professionals’ mental health and well-being during the COVID-19 outbreak. The same results were found in previous studies in which individuals with a history of mental health disorders are more vulnerable to stressful incidents, which usually lead to worse mental health and well-being.^16,17^ The COVID-19 outbreak is much more difficult for medical professionals with mental health disorders.

This research found that depression and anxiety among medical professionals is affected by social media exposure. Nowadays, social media are often seen as fast and effective platforms for searching, sharing, and distributing information.^18^ However, higher daily social media use was associated with greater dispositional anxiety and increased likelihood of having a probable anxiety disorder. The problematic effect of social media to mental health, probably due to an elevated stress level induced by a flood of unfiltered information, rumors and a more emotional style of communication. The World Health Organization (WHO) created the term “infodemic” in the context of the COVID-19 crisis for an “over-abundance of information—some accurate and some not—that makes it hard for people to find trustworthy sources and reliable guidance when they need it.”^19^ Social media amplifies the “infodemic” by making it go faster and further.^20^ A recent research revealed that the frequency of social media exposure is significantly positively correlated to symptoms of depression and anxiety within the general population in China during the pandemic outbreak.^21^

This research found that depression and anxiety among medical professionals is affected by social media exposure. These results may attribute to the confusion raised by “infodemic” from social media, particularly once a critical mass of predominantly negative news or unreliable information is reached. On the other hand, medical professionals with psychological disorders or some pathological personality traits tend to be more sensitive to negative information about the disaster, such as pandemic outbreak.^22^ Thus, exposure to a preponderance of negative information about pandemic and misinformation from the social media may have a negative impact on their mental health, which may lead to a vicious circle, thereby becoming vulnerable to negative emotions, such as depression and anxiety.^23,24^ Given the proliferation of social media, identifying the mechanisms and direction of this association is critical for informing interventions that address the impact of confusion on anxiety and depression among medical professionals. It is important to protect medical professionals from confusing information to reduce the risk of anxiety and depression.

In addition, somatic discomfort triggered by social media is largely unreported. Somatic experiences are a primary response of autonomic nervous system reactivity to stressful information or incidents. Previous research found that maladaptive patterns of autonomic nervous system reactivity are putative risk factors underlying the development of psychopathology. Several studies found that sympathetic reactivity or autonomic nervous system dysregulation measured after trauma or stressful incidents has been associated with an increased likelihood of developing psychopathology across a range of domains, including posttraumatic stress disorder, depression, and anxiety problems.^25,26^ Busso and his colleagues discovered that the interplay between the degree of social media exposure to traumatic information, preattack psychopathology, and autonomic nervous system reactivity predicted PTSD symptom onset following the terrorist attack at the 2013 Boston Marathon.^27^ Our study further found that somatic discomfort triggered by social media and a history of mental health disorders are correlated with the onset of anxiety and depression symptoms during the COVID-19 outbreak. The results of this research suggest that it is very important to reduce allostatic load in stress response systems, and previous research found that yoga-based practices, meditation, and relaxation techniques are effective ways to address the dysfunction of autonomic nervous system dysfunction, which may be an effective way to cope with the somatic discomfort triggered by social media.^28,29^

## Conclusions

The mental health of medical staff is in poor condition, with approximately 30% of medical professionals having a history of mental illness. To prevent medical professionals from experiencing psychological disturbances during the COVID-19 outbreak, it is important to provide adequate psychological assistance for medical professionals with mental health diseases. Health services and promotion should optimize the influence of social media on medical professionals to provide and enhance effective psychological strategies or interventions to improve their mental health and well-being.

### Limitations

It is important to note that only 1 item was used to evaluate somatic discomfort and confusion triggered by social media, thus limiting the generalizability of the current results. Second, somatic discomfort is a subjective experience that may not fully reflect an individual’s physical response to exposure to information from social media, and it is important for these results to be replicated in the future using experimental designs to test the autonomic nervous system reactivity among medical workers. Third, the current research does not inquire about the work role, workload, and working environment of medical personnel. Our research aimed to focus on social media and the history of mental illness for current anxiety and depression. Future studies will focus on the role of occupation, workload, and working environment on psychological disturbances. Additionally, the current study was a cross-sectional design; therefore, no causal conclusions can be drawn. Our findings should not be generalized to a broader population.
